# Prediction Model of Three-Dimensional Machined Potassium Dihydrogen Phosphate Surfaces Based on a Dynamic Response Machining System

**DOI:** 10.3390/ma15249068

**Published:** 2022-12-19

**Authors:** Qilong Pang, Jianlong Xiong

**Affiliations:** College of Mechatronics Engineering, Nanjing Forestry University, Nanjing 210037, China

**Keywords:** surface topography, machining process, potassium dihydrogen phosphate, dynamic response

## Abstract

To comprehensively obtain the effect of the machining process on the three-dimensional surface topography of machined potassium dihydrogen phosphate crystals, a dynamic response model of a machining system was built to calculate the dynamic displacement variables in the different processing directions. This model includes almost all processing factors, such as cutting parameters, environment vibration, radial and axial runout of the spindle, cutting tool parameters, material parameters, guide way error, fast tool servo and lubrication condition errors, etc. Compared with the experimental results, the three-dimensional topographies and two-dimensional profiles of the simulation surfaces were nearly consistent with those of experimental machined surfaces. As the simulation shows, the cutting parameters, axial runout of the spindle, and the output noise of the fast tool servo can respectively impact the main, low, and high frequencies of the machined surface topography. The main frequency of all the simulated and experimental surfaces in this study was 0.0138 μm^−1^. The low and high frequencies of the simulation surfaces had slight differences, about 0.003 μm^−1^ from those of the experimental surfaces. The simulation model, based on dynamic response, can accurately predict the entire machining process and three-dimensional topographies of machined potassium dihydrogen phosphate surfaces.

## 1. Introduction

In the fields of high-power laser systems and inertial confinement fusion, potassium dihydrogen phosphate (KH2PO4, KDP) crystal is widely used as the key material for nonlinear frequency-doubled components [1,2]. Machined KDP surface topographies can impact the optical performance of KDP components [3,4]. Therefore, mechanism analysis for the forming of machined surface topography is useful for improving machining quality and enhancing the optical performance of KDP components. The forming of machined surface topography is directly related to the machining process. All factors in the machining process, including the various vibrations of the cutter, workpiece, spindle, guide way, and the processing parameters such as feed rate, cutting speed, cutting depth, lubrication, material parameters, and the geometric parameters of the cutting tool can act on the machined surface topography [5,6].

Many experimental studies have been carried out that show that the conditions of the machining process can directly impact the topographies and performances of machined surfaces. Frequency or waviness features in the machined surfaces are the main aspects affected by the various machining process factors [7]. The cutting parameters under dry conditions and a minimum quantity of lubrication were compared to analyze their influence on surface roughness and topographies [8,9]. Lubrication conditions between tool and material, the ambient temperature, and the cutting parameters of the machining process could affect the surface roughness of machined material [10]. A good level of lubrication and cooling in the process of machining could obviously improve the surface quality of the machined surface [11]. The cutting parameters, such as feed rates, cutting speeds, and cutting depth were shown to have a direct influence on machined surface roughness, two-dimensional (2D) profile, and surface topography [12]. Topographies of the machined surface could severely impact the optical performance of the KDP components. Scratches in machined KDP surfaces could impact the laser-damage threshold of KDP components [13]. The main frequency features in KDP crystal surfaces machined using the fly cutting method could reduce optical field performance [14]. The frequency features of different wavelengths machined using the micro-milling method had different influences on the optical performance of KDP crystals [15]. To obtain the effects of surface topography on the performance of machined KDP crystals, the important prerequisite is to analyze the forming mechanism of the machined surface topography.

Some researchers have built theoretical models to predict machined surface topographies. The response surface method is used to verify the relation between the cutting parameters and surface roughness in the turning process [16,17]. The surface topography machined by abrasive belt grinding was numerically calculated based on the Johnson transformation system and a filter impulse function [18]. A least squares support vector machine-based algorithm was developed to predict surface roughness in machined surfaces [19]. A combination of a finite element model and cellular automata model was used to predict microstructure in the machined surface based on the material model [20]. The integration methods of fast Fourier, discrete wavelet, and discrete shearlet transforms were used to predict surface roughness on machined surfaces in [21]. Machined surface errors and behavior of materials have been simulated and calculated using a dynamic fixture-workpiece system based on finite element analysis [22,23]. The regression method and an artificial neural network were used to predict three-dimensional (3D) surface topographies based on cutting parameters and cutter vibration [24]. The 3D form errors of machined surfaces were evaluated and analyzed using the integration of the unified Jacobian–Torsor model and skin model shapes [25]. Recently, machine learning has been an efficient method for predicting and evaluating the relationship between machined surface characteristics and the machining process. Machine learning based on cutting force variation modeling was used to improve machined surface shape prediction [26]. Machined surface roughness and 2D profiles of wire electrical discharge machining were also predicted by machine learning algorithms [27].

In the above predictions of machined surface topographies, the study subjects of most studies were the 2D profiles and their roughness. Compared with 3D surface topographies, 2D profiles cannot completely reflect the topographic information of the machined surfaces. For the above studies, the machining process factors that were used to analyze the machined surface topographies were mainly cutting parameters, material parameters, lubrication conditions, and vibration of the cutter. However, from the systematic perspective, other factors such as environment vibration, runout of the spindle, tool wear, the geometric parameters of the cutting tool, the output noise of the fast tool servo, etc., could also impact the topographies of machined surfaces. The purpose of this investigation was to comprehensively reveal the relationship between the machining process and the 3D surface topographies of machined KDP crystals. To do this, this study provides a dynamic response model, which includes almost all the factors of the machining system to predict the 3D surface topographies of machined KDP crystals. With this prediction model, the entire process of machining KDP is simulated to optimize machining parameters and achieve the optimum surface topography to enhance the optical performance of the machined KDP crystals.

## 2. Experiments and Methods

### 2.1. Experiments

**Figure 1:**
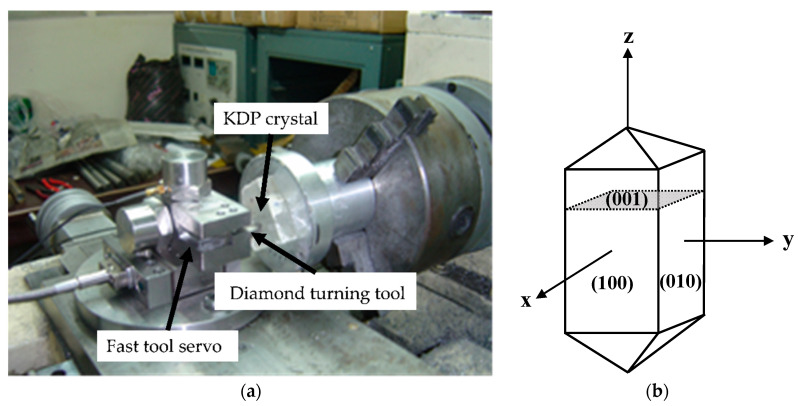
The KDP crystal machining experiment: (**a**) cutting experiment; (**b**) position of the (001) face in the KDP crystal.

**Figure 2:**
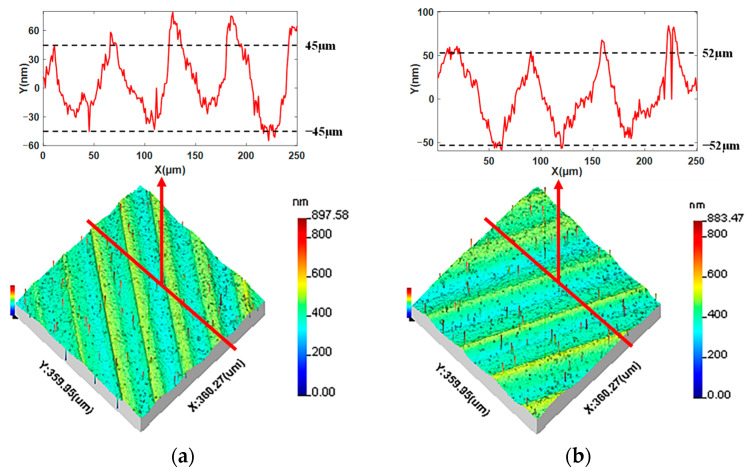
Experimental 3D surface topographies and 2D profiles: (**a**) experiment surface with a_p_ = 6 μm, n = 1300 r/min, f = 12 μm/r; (**b**) experiment surface with a_p_ = 9 μm, n = 1400 r/min, f = 14 μm/r.

The (001) faces of KDP crystals with a size of 6 cm × 6 cm were machined using the face turning method shown in Figure 1. In the cutting experiments, the fast tool servo was self-made, and the machine tool was a CXM6125 (range of spindle speed 63–3150 r/min; range of feed rate 5–100 μm/r) made by Jinan First Machine Tool Company. The machining process was dry cutting, without lubrication or coolant. Because KDP is a somewhat soft-brittle material, there is no built-up edge in the machining process. The material of the cutting tool was diamond and its parameters are listed in Table 1. To verify the prediction model, two experimental surfaces were machined using the approximate cutting parameters; the cutting results are shown in Figure 2. The cutting parameters used in the experiments are shown in Table 2. The mechanical parameters of the KDP crystal are listed in Table 3 and were used in the simulation model. The environment temperature during the machining of the KDP was 23 °C. The results of the experiments are repeatable with the same processing parameters.

The surface topographies of the machined KDP crystals were measured using a Taylor Surf CCI white light interferometer. The measurement sampling area was 360 μm × 360 μm; the number of sampling points was 256 × 256. The original measurement results are shown in Figure 2. In order to clearly compare with the simulation results, the 2D profiles of the different experiments are extracted and shown in Figure 2. It can be observed that the 2D profiles in Figure 2a have 5 and 4 peaks and their amplitude is about 45 μm and 52 μm, respectively.

### 2.2. Simulation Models

**Figure 3:**
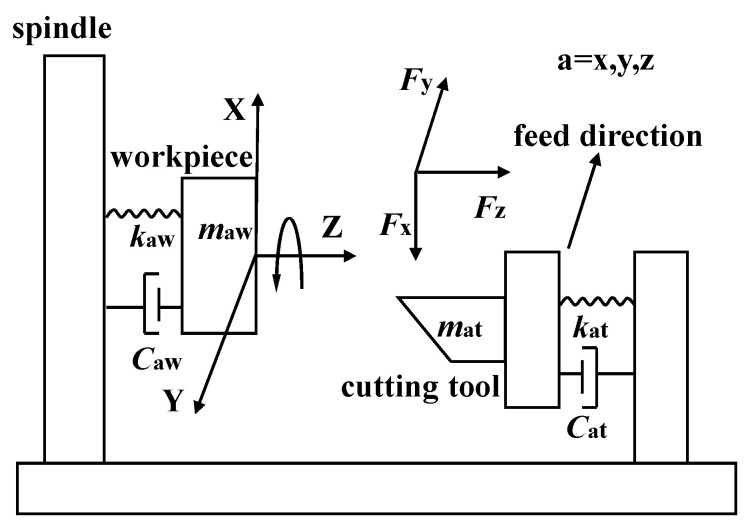
Machining process simulated by the dynamic response model.

The machining process shown in Figure 3 was simulated using the dynamic response model and was consistent with the experiment process. The coordinate system and the directions of feed and spindle rotation in Figure 3 were adopted in the simulation model. The cutting, cutting tool, and material parameters in the simulation were equal to those used in the experiments.

**Figure 4:**
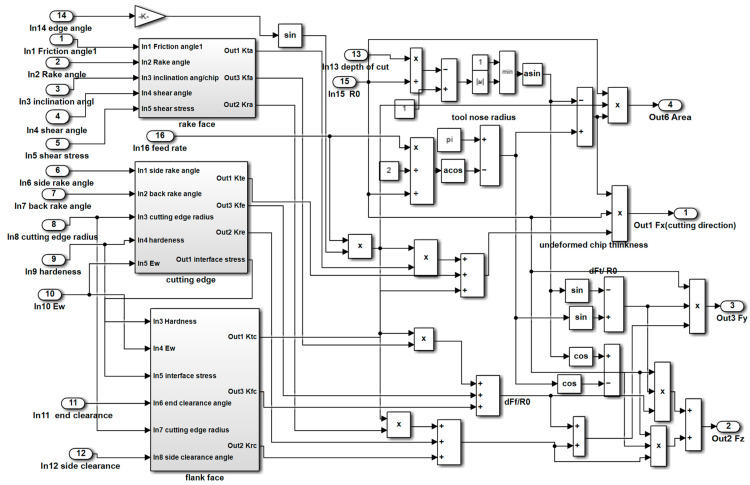
Simulink model of the dynamic cutting forces.

In this study, the machining system shown in Figure 3 was simplified to a second order elastic-damping vibration system. The dynamic cutting forces between the cutting tool and workpiece induce their dynamic displacements. According to the mass, damping, and stiffness of the cutting tool and workpiece system, the whole machining system can be described as the functions of the dynamic cutting forces in the *X*, *Y,* and *Z* directions, and shown as

(1) $$\left\{ \begin{array}{l} m_{xt}{\overset{..}{x}}_{t}(t)+c_{xt}{\dot{x}}_{t}(t)+k_{xt}x_{t}(t)=F_{x}(t)\\ m_{yt}{\overset{..}{y}}_{t}(t)+c_{yt}{\dot{y}}_{t}(t)+k_{yt}y_{t}(t)=F_{y}(t)\\ m_{zt}{\overset{..}{z}}_{t}(t)+c_{zt}{\dot{z}}_{t}(t)+k_{zt}z_{t}(t)=F_{z}(t)\\ m_{xw}{\overset{..}{x}}_{w}(t)+c_{xw}\dot{x_{w}}(t)+k_{xw}x_{w}(t)=-F_{x}(t)\\ m_{yw}\overset{..}{y_{w}}(t)+c_{yw}\dot{y_{w}}(t)+k_{yw}y_{w}(t)=-F_{y}(t)\\ m_{zw}\overset{..}{z}w(t)+c_{zw}{\dot{z}}_{w}(t)+k_{zw}z_{w}(t)=-F_{z}(t) \end{array}\right.$$

where *m_at_* and *m_aw_* (*a* = *x*,*y*,*z*) are the equivalent mass of the cutting tool and workpiece in the *X*, *Y*, and *Z* directions; *c_at_* and *c_aw_* (*a* = *x*,*y*,*z*) are the equivalent damping of the cutting tool and workpiece in the *X*, *Y,* and *Z* directions; *k_at_* and *k_aw_* (*a* = *x*,*y*,*z*) are the equivalent stiffness of the cutting tool and workpiece in the *X*, *Y,* and *Z* directions; *x_t_*, *y_t_*, and *z_t_* are the dynamic displacements of the cutting tool in the *X*, *Y,* and *Z* directions, respectively; *x_w_*, *y_w_*, and *z_w_* are the dynamic displacements of the workpiece in the *X*, *Y,* and *Z* directions, respectively; and *F_x_*, *F_y_*, and *F_z_* are the dynamic cutting forces in the *X*, *Y,* and *Z* directions. The Simulink (version 2012a) model of the dynamic cutting forces is shown in Figure 4.

**Figure 5:**
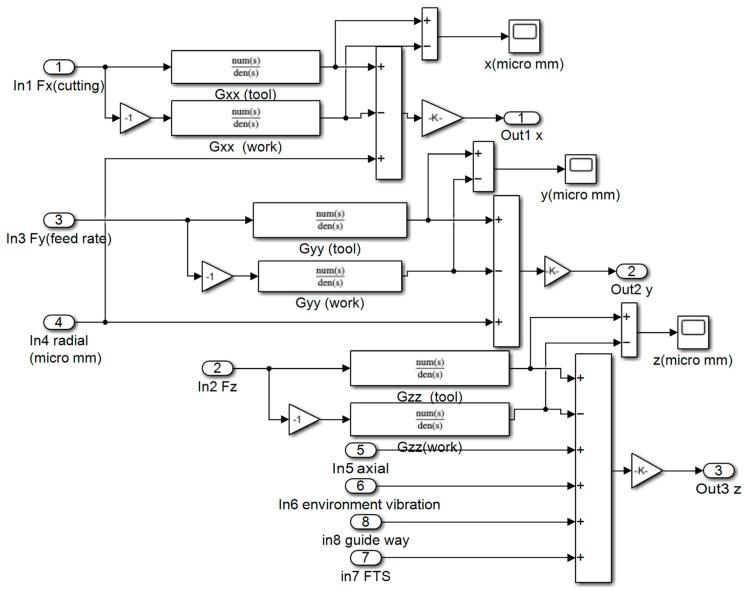
Simulink model of the dynamic displacements of the machined surface.

The purpose of the simulation was to obtain the dynamic displacements as the machined surfaces. The dynamic displacements of the machined surface in the *X*, *Y,* and *Z* directions correspond to the difference value between two dynamic displacements of the cutting tool and workpiece, calculated as

(2) $$\left\{ \begin{array}{l} detx_{i,j}=x_{t}-x_{w}\\ {dety}_{i,j}=y_{t}-y_{w}\\ detz_{i,j}=z_{t}-z_{w} \end{array}\right.\;\left(i=1,2,\cdots ,M;j=1,2,\cdots ,N\right)$$

where *detx_i,j_*, *dety_i,j_*, and *detz_i,j_* are the dynamic displacements of the machined surface in the *X*, *Y,* and *Z* directions, respectively; *M* is the number of spindle rotations in the machining process; and *N* is the number of sampling points per revolution. The Simulink model of the dynamic displacement is shown in Figure 5. In Figure 5, Out1, Out2, and Out3 are the dynamic displacements in the *X*, *Y*, and *Z* directions, respectively, and In1, In3, and In2 are the dynamic cutting forces in the *X*, *Y*, and *Z* directions, respectively. 

**Figure 6:**
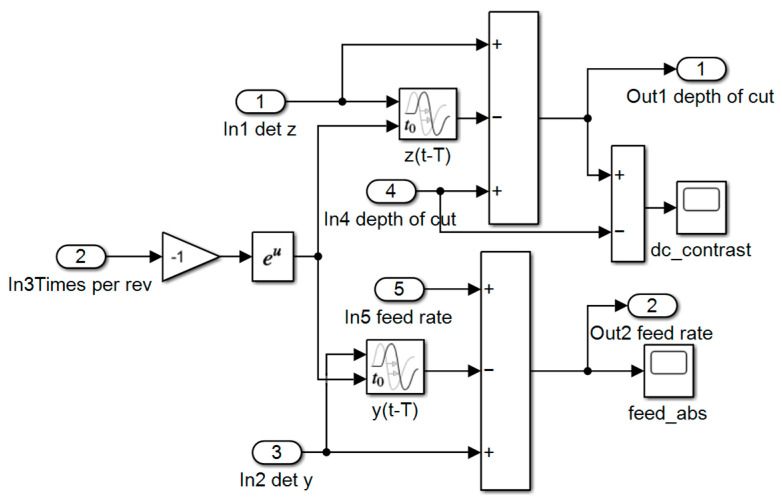
Simulink model of regenerative vibration.

Regenerative vibration presents as variation in cutting thickness and width, which then impacts the cutting force. It is a process of cyclical generation and can be defined as

(3) $$\left\{ \begin{array}{l} detc_{t}=z(t)-z(t-T)\\ detc_{w}=y(t)-y(t-T) \end{array}\right.$$

where *detc_t_* is the variation in cutting width; *detc_w_* is the variation in cutting thickness; *z*(*t*) is the cutting depth at the current rotation; *y*(*t*) is the feed rate at the current rotation; and *T* is the delay time. The Simulink model of regenerative vibration is shown in Figure 6. In Figure 6, we see that the regenerative vibration can induce variation in cutting depth and feed rate, and the changed cutting depth and feed rate are output as Out1 and Out2.

**Figure 7:**
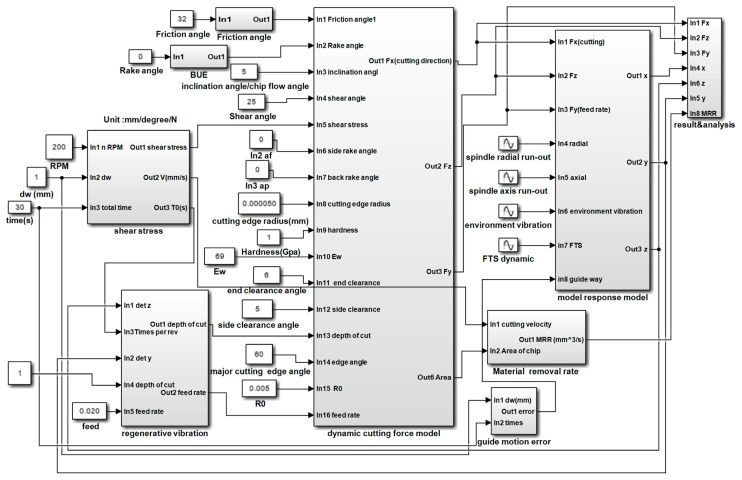
Complete Simulink model of the dynamic response.

The complete Simulink model of the dynamic response is shown in Figure 7. In the complete model, the machining process system integrates the dynamic cutting force, dynamic displacement, regenerative vibration, and shear stress modules. The shear stress module is a simple function of square wave, and the Simulink model of it not necessary to show. In Figure 7, it can be observed that the input variables of the Simulink model contain all aspects of machining process and the output results are the dynamic cutting forces and displacements of the machined surface.

In the simulation, almost all factors in the machining process were considered as input variables of the Simulink model. In the model, the values for built-up edge and coolant are zero; the parameters for the tool, cutting, and KDP crystal are shown in Table 1, Table 2 and Table 3. The other input variables of the simulation model were the various vibrations in the machining process and defined as usual values for general machine tools. These input variables are listed in Table 4 and Table 5. The mass, damping, and stiffness values of the tool and workpiece systems were obtained using the dynamic experiments and are shown in Table 6.

To verify the simulation’s prediction for the frequency features of the machined surfaces, the power spectrum density (PSD) method was used to compare the frequency features of the experimental and simulated results, shown as [28]

(4) $$PSD\left(f\right)=\underset{L\to \infty }{\lim}\frac{{\left|z\left(f,L\right)\right|}^{2}}{L}$$

where *L* is sampling length, *f* is the spatial frequency, and *z*(*f*, *L*) is the Fourier transform of the 2D surface profile.

## 3. Results and Discussion

**Figure 8:**
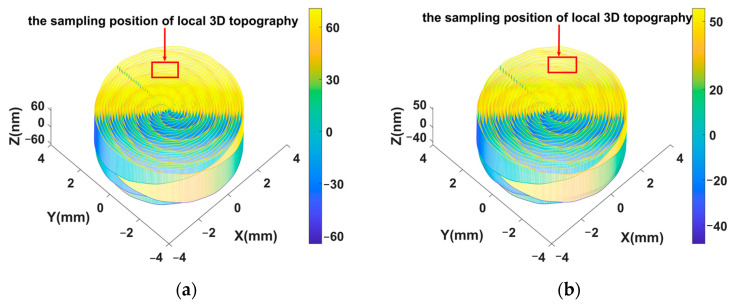
Entire 3D topographies of simulated results: (**a**) simulated surface with ap = 9 μm, n = 1300 r/min, and f = 12 μm/r; (**b**) simulated surface with ap = 6 μm, n = 1400 r/min, and f = 14 μm/r.

In order to reduce the computation, the radius of the workpiece in the turning simulations was 3.6 mm. The entire machining process of the workpiece was simulated and its 3D surface topographies are shown in Figure 8. It can be observed that the obvious waviness textures around the center of the workpiece are distributed in the machined surfaces. The maximum height of the entire simulated surface for the input parameters ap = 9 μm, n = 1300 r/min, and f = 12 μm/r was about 60 nm and higher than the other surface. To clearly compare the results of the experiment and simulation, local 3D topographies with an area of 360 μm × 360 μm were extracted from the entire simulation surface.

**Figure 9:**
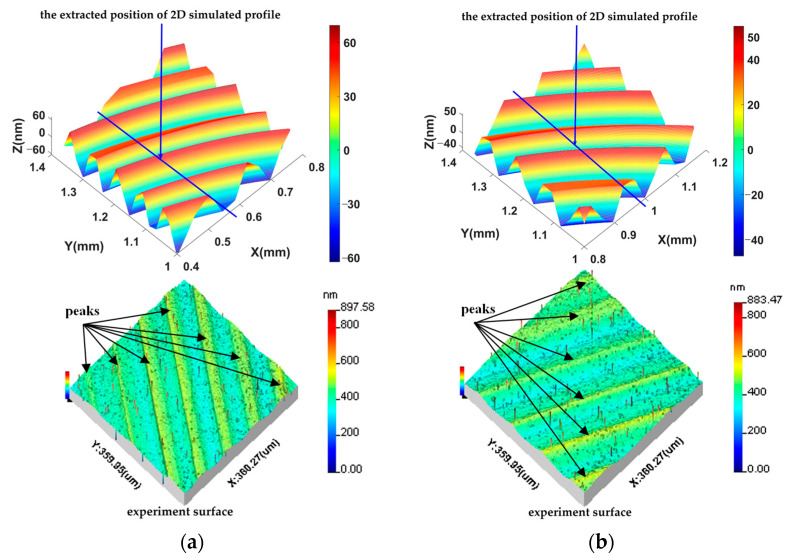
Comparison between the local 3D topographies of simulation results and experiment surfaces: (**a**) results for a_p_ = 9 μm, n = 1300 r/min, and f = 12 μm/r; (**b**) results with a_p_ = 6 μm, n = 1400 r/min, and f = 14 μm/r.

The 3D simulation surfaces in Figure 8 can reflect the surface roughness and geometric error of machined surfaces and are similar to the surface texture of machined KDP components. However, the weakness of the entire simulation surface is that it cannot clearly reflect the details of 3D topography. The extraction positions of the local 3D topographies are pointed out in Figure 8, and the local 3D simulation and experiment results are presented in Figure 9 to compare the details of the 3D surface topographies. Comparing the two results, it can be seen that there are seven and six clear peaks in the simulation, shown in Figure 9a,b respectively. The comparison of the two results reveals that the distributions of peaks and wavelengths in the simulation surfaces are accordant with those in the experiment surfaces. The amplitude of local 3D topographies for the input parameters ap = 9μm, n = 1300 r/min, and f = 12 μm/r was about 10 nm higher than that with cutting parameters of ap = 6 μm, n = 1400 r/min, and f = 14 μm/r; this is consistent with the experiment results and accords with the cutting theory. It can be found that the distribution directions of waviness in the results are different. This is induced by differences in sampling position in the experiment and simulation surfaces. However, in general the entire simulation surface maintains the directional features of the machined surface.

**Figure 10:**
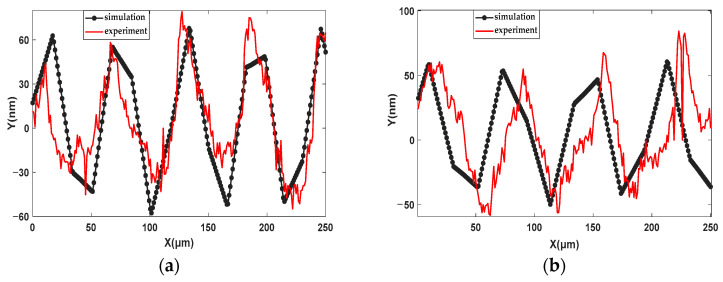
Comparison between the surface profiles of simulation results and experiment surfaces: (**a**) results with ap = 9 μm, n = 1300 r/min, and f = 12 μm/r; (**b**) results with ap = 6 μm, n = 1400 r/min, and f = 14 μm/r.

To further analyze the effects of the dynamic response model on the details of the machined KDP surface topography, the 2D simulation surface profiles were extracted from the simulated local 3D topographies; the extracted positions are shown in Figure 9 and the extraction results of the 2D profiles are shown in Figure 10. Comparing the results, it can be seen that there are five and four recognizable peaks in the simulation and experiment, as shown in Figure 10a,b, respectively. The peak number of the simulation profiles is equal to that of the experiment profiles. From Figure 10, it can be inferred that the average interval distance of peaks in the simulation profile with the parameters of ap = 9 μm, n = 1300 r/min, and f = 12 μm/r was 55 μm, and that of the other simulation profile was 66 μm. The average interval distances of peaks in the simulation profiles were consistent with the experiment results. The amplitude of the 2D simulation profile with input parameters of ap = 9 μm, n = 1300 r/min, and f = 12 μm/r was 60 nm and about 10 nm higher than that with cutting parameters of ap = 6 μm, n = 1400 r/min, and f = 14 μm/r. The amplitudes of the 2D simulation profile are very comparable to those of the experiment profiles. The distinct difference between the experiment and simulation profiles in Figure 10 is that many micro-waves are overlapped with the main frequencies in the experiment profiles and the simulation profiles are smoother. The reasons for the forming of micro-waviness in the experiment profiles is that brittle fractures in the KDP occurred during machining as well as instrument noise in measurement.

**Figure 11:**
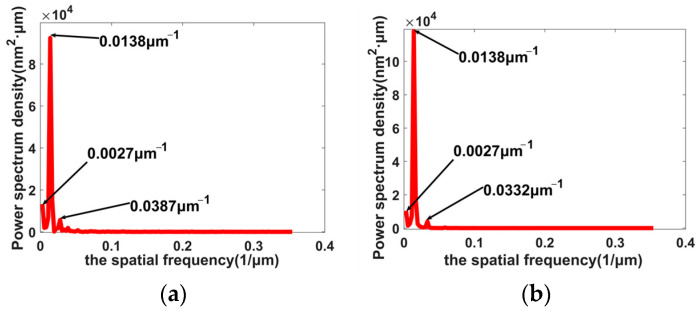

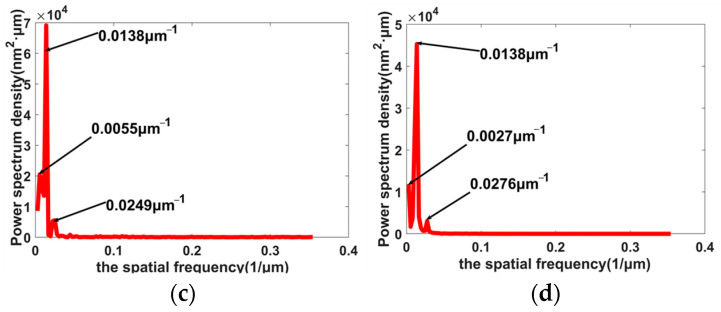
PSD of the simulation and experiment profiles: (**a**) PSD of experiment profiles with ap = 9 μm, n = 1300 r/min, and f = 12 μm/r; (**b**) PSD of simulation profiles with ap = 9 μm, n = 1300 r/min, and f = 12 μm/r; (**c**) PSD of experiment profiles with ap = 6 μm, n = 1400 r/min, and f = 14 μm/r; (**d**) PSD of simulation profiles with ap = 6 μm, n = 1400 r/min, and f = 14 μm/r.

There are various vibrations, such as the runout of the spindle, environment vibration, the noise of the fast tool servo, etc., in the machining process. These vibrations can be reflected in the machined surfaces. To analyze the effect of processing frequency factors on the frequency features of machined surfaces, the PSD values of the simulation and experiment profiles in Figure 10 were calculated and are shown in Figure 11. From the results of the PSD analysis, the main frequency of all profiles was 0.0138 μm^−1^. In general, the main frequency of the machined surface was mainly influenced by the cutting parameters. Because the feed rates of 12 μm/r and 14 μm/r are very close, the main frequencies of all profiles were unchanged. Through many attempts to change the input parameters of various vibrations, it was found that the axial runout of the spindle and the output noise of the fast tool servo can respectively impact the low and high frequencies of the simulation surfaces. For the profiles with cutting parameters of ap = 9 μm, n = 1300 r/min, and f = 12 μm/r, the low frequencies of experiment and simulation profiles were 0.0027 μm^−1^, and this shows that the axial runout of the spindle defined in the dynamic response model was nearly the same as that in the experiment. The high frequencies of the experiment and simulation profiles were 0.0387 μm^−1^ and 0.0332 μm^−1^, respectively, and this shows that the output noise of the fast tool servo in the simulation was slightly different from that in the experiment. For the profiles with cutting parameters of ap = 6 μm, n = 1400 r/min, and f = 14 μm/r, the low and high frequencies of the experiment and simulation profiles were 0.0055 μm^−1^, 0.0249 μm^−1^, 0.0027 μm^−1^, and 0.0276 μm^−1^; the differences of the low and high frequencies were about 0.003 μm^−1^ between the experiment and simulation profiles. The difference also suggests that the axial runout of the spindle and output noise of the fast tool servo are not constants and changed with the variation of cutting parameters.

In this study, the runout of the spindle and the output noise of the fast tool servo were input to the simulation model as constants, and this created slight errors in the prediction of frequency features in the machined surfaces. Further research should be undertaken to investigate the effects of cutting parameters on the spindle runout and output noise of the fast tool servo.

## 4. Conclusions

The results of this study lead to the following conclusions:

(1) In summary, these simulation results show that the dynamic response model built in this study includes almost all machining process factors as the input parameters and can predict the machined KDP surface topography at both macro- and micro-scales. The simulation model presented in this study can also be used to predict the face turning-machined surfaces of other materials when the material parameters are changed.
(2) The obvious waviness textures around the center of the workpiece are distributed in the entire 3D simulation surface. The entire simulation surface can represent the whole machining process of the complete workpiece and reflects the surface roughness and geometric error of machined KDP surfaces; The 3D entire simulation surface maintains the directional features of the machined surfaces.
(3) The amplitudes and wavelengths of 3D spatial frequencies in the local 3D simulated topographies are accordant with those in the experiment surfaces. The differences in sampling position induce the different distribution directions of waviness in the local 3D simulated topographies and experiment surfaces.
(4) The amplitudes and interval distances of peaks in the simulation profiles are consistent with those in the experiment profiles. The simulation profiles are smoother than the experiment profiles. The micro-waviness that overlaps in the experiment profiles is due to brittle fractures of the KDP crystal and measurement noise.
(5) The simulation and experiment surfaces have the same main frequencies. Their low and high frequencies have slight differences. The reason for this is that the runout of the spindle and output noise of the fast tool servo are not constants and changed with variation in cutting parameters.

## Figures and Tables

**Table 1:** The tool parameters used in the experiments.

Parameters	Values
Tool tip radius *r_ε_* (mm)	3.2
Tool edge radius *r_n_* (nm)	150
Rake angle *γ*_0_ (°)	−10
Clearance angle *α*_0_ (°)	9
Roughness of rake face *R*_a_ (nm)	5.4
Roughness of flank face *R*_a_ (nm)	4.3
Roughness of cutting edge *R*_a_ (nm)	7.6

**Table 2:** Cutting parameters used in the experiments.

Cutting Experiment	Cutting Depth a_p_/μm	Spindle Speed n/(r/min)	Feed Rate f/(μm/r)
1	9	1300	12
2	6	1400	14

**Table 3:** Mechanical parameters of the KDP crystal.

Parameters	Values
Density (kg/m^3^)	2338
Elastic modulus (GPa)	75
Poisson ratio	0.33
Mohs hardness	2.5
Yield strength (MPa)	240

**Table 4:** Input vibration variables of the simulation model.

Input Vibration Variables	Amplitude/μm	Frequency/Hz
environment vibration	0.01	500
radial runout of the spindle	0.05	30
axial runout of the spindle	0.05	30
output noise of fast tool servo	0.02	125

**Table 5:** Motion errors of the machine tool guide.

Input Variable	Error in the Z Direction/μm	Error in the Z Direction/μm
motion error of guide	15	40

**Table 6:** Mass, damping, and stiffness of tool and workpiece.

Parameters	Tool System			Workpiece System		
	X	Y	Z	X	Y	Z
equivalent mass (Kg)	74.3			1045.4		
static stiffness (N/μm)	164	143	167	135	124	156
damping ratio	0.82	0.74	0.85	0.64	0.56	0.68

## Data Availability

Not applicable.

## References

[1] Zhang Z., Wang H., Quan X., Pei G., Tian M., Liu T., Long K., Li P., Rong Y. (2018). Optomechanical analysis and performance optimization of large-aperture KDP frequency converter. Opt. Laser. Technol..

[2] Spaeth M., Manes K., Kalantar D. (2016). Description of the NIF Laser. Fusion Sci. Technol..

[3] Lza B., Swa B., Hy C. (2021). Study on optical performance and 532 nm laser damage of rapidly grown KDP crystals. Opt. Mater..

[4] Baig M., Anis M., Muley G. (2017). Comprehensive study on crystal growth, optical and dielectric properties of potassium dihydrogen orthophosphate crystal influenced by organic additive salicylic acid. Optik.

[5] Bei G., Ma C., Wang X., Sun J., Ni X. (2022). On the optimal texture shape with the consideration of surface roughness. Sci. Rep..

[6] Bei G., Ma C., Wang X., Sun J., Ni X. (2022). Study on Tribological Characteristics of Textured Surface under Convergent Oil Film Gap. Lubricants.

[7] Adamczak S., Zmarzły P. (2017). Influence of raceway waviness on the level of vibration in rolling-element bearings. Bull. Pol. Acad. Sci. Technol..

[8] Mehmet E.K., Munish K.G., Mehmet B., Nafiz Y., Grzegorz M.K., Mustafa G. (2021). Influence of duplex jets MQL and nano-MQL cooling system on machining performance of Nimonic 80A. J. Manuf. Process..

[9] Sen B., Mia M., Krolczyk G.M. (2021). Eco-Friendly Cutting Fluids in Minimum Quantity Lubrication Assisted Machining: A Review on the Perception of Sustainable Manufacturing. Int. J. Precis. Eng. Manuf. Green Technol..

[10] Munish K.G., Niesłony P., Sarikaya M., Mehmet E.K., Kuntoğlu M., Jamil M. (2022). Tool wear patterns and their promoting mechanisms in hybrid cooling assisted machining of titanium Ti-3Al-2.5V/grade 9 alloy. Tribol. Int..

[11] Şap S., Usca Ü.A., Uzun M., Kuntoğlu M., Salur E., Pimenov D.Y. (2022). Investigation of the effects of cooling and lubricating strategies on tribological characteristics in machining of hybrid composites. Lubricants.

[12] Emin S. (2022). Understandings the tribological mechanism of Inconel 718 alloy machined under different cooling/lubrication conditions. Tribol. Int..

[13] Yang H., Cheng J., Liu Z., Liu Q., Zhao L., Wang J., Chen M. (2020). Dynamic behavior modeling of laser-induced damage initiated by surface defects on KDP crystals under nanosecond laser irradiation. Sci. Rep..

[14] Cheng J., Xiao Y., Liu Q., Yang H., Zhao L., Chen M., Tan J., Liao W., Chen J., Yuan X. (2018). Effect of surface scallop tool marks generated in micro-milling repairing process on the optical performance of potassium dihydrogen phosphate crystal. Mater. Des..

[15] Miao J., Yu D., An C., Ye F., Yao J. (2017). Investigation on the generation of the medium-frequency waviness error in flycutting based on 3D surface topography. Int. J. Adv. Manuf. Technol..

[16] Xiao M., Shen X., Ma Y. (2018). Prediction of Surface Roughness and Optimization of Cutting Parameters of Stainless Steel Turning Based on RSM. Math. Probl. Eng..

[17] Raj A., Wins K.L.D., Varadarajan A.S. (2019). Optimization of cutting parameters and prediction of surface roughness during hard turning of H13 steel with minimal vegetable oil based cutting fluid application using response surface methodology. IOP Conf. Ser.: Mater. Sci. Eng..

[18] Zou L., Liu X., Huang Y., Fei Y. (2019). A numerical approach to predict the machined surface topography of abrasive belt flexible grinding. Int. J. Adv. Manuf. Tech..

[19] Zhang N., Shetty D. (2016). An effective LS-SVM-based approach for surface roughness prediction in machined surfaces. Neurocomputing.

[20] Liu H., Zhang J., Xu B., Xu X., Zhao W. (2020). Prediction of microstructure gradient distribution in machined surface induced by high speed machining through a coupled FE and CA approach. Mater Design.

[21] Prabhakar D., Kumar M., Krishna G. (2020). A Novel Hybrid Transform approach with integration of Fast Fourier, Discrete Wavelet and Discrete Shearlet Transforms for prediction of surface roughness on machined surfaces. Measurement.

[22] Dong Z., Jiao L., Wang X., Liang Z., Liu Z., Yi J. (2016). FEA-based prediction of machined surface errors for dynamic fixture-workpiece system during milling process. Int. J. Adv. Manuf. Tech..

[23] Jia X., Zhou Y., Wang Y. (2022). Deformation behavior and constitutive model of 34CrNi3Mo during thermo-mechanical deformation process. Materials.

[24] Lin Y., Wu K., Shih W., Hsu P., Hung J. (2020). Prediction of surface roughness based on cutting parameters and machining vibration in end milling using regression method and artificial neural network. Appl. Sci..

[25] Yi Y., Liu T., Yan Y., Feng J., Ni Z., Liu X. (2022). A novel assembly tolerance analysis method considering form errors and partial parallel connections. Int. J. Adv. Manuf. Technol..

[26] Shao C., Ren J., Wang H., Jin J., Hu S. (2017). Improving machined surface shape prediction by integrating multi-task learning with cutting force variation modeling. J. Manuf. Sci. Eng..

[27] Ulas M., Aydur O., Gurgenc T., Ozel C. (2020). Surface roughness prediction of machined aluminum alloy with wire electrical discharge machining by different machine learning algorithms. J. Mater. Res. Technol..

[28] Itoh T., Yamauchi N. (2007). Surface morphology characterization of pentacene thin film and its substrate with under-layers by power spectral density using fast Fourier transform algorithms. Appl. Surf. Sci..

